# Adaptation and Testing of the Factorial Structure of the Physical Education Grit Scale for Use in Secondary Education in Spain

**DOI:** 10.3390/ijerph191610008

**Published:** 2022-08-13

**Authors:** Jerónimo González-Bernal, Sergio Gonzalez-Bernal, Carlos Salavera, Carmen Fernández-Ortega, Rubén Trigueros Ramos, José M. Aguilar-Parra, María Josefa González-Santos

**Affiliations:** 1 Department of Psychology, University of Burgos, 09001 Burgos, Spain; 2 Department of Psychology, University of Zaragoza, 50009 Zaragoza, Spain; 3 Hum-878 Research Team, Health Research Centre, Department of Psychology, University of Almería, 04120 Almeria, Spain

**Keywords:** grit, psychometric properties, physical education, adolescents

## Abstract

The challenges faced by students during Physical Education classes embrace both physical and academic aspects. Therefore, each individual possesses a series of internal psychological mechanisms, such as Grit, which allow them to adapt and overcome the vicissitudes. However, there are no scales that assess Grit in Span. Thus, the aim of the present study is to test the factor structure of the Physical Education Grit Scale in Span of Physical Education classes. For this purpose, 857 secondary school students took part in the present study. In order to test the factor structure, a confirmatory factor analysis, an exploratory factor analysis, an analysis of the reliability of the questionnaire and an analysis of temporal stability were carried out. The results showed that the factor structure consisted of two factors and four sub-factors (χ^2^/df = 2.17 (*p* = 0.001); CFI = 0.96; TLI = 0.96; IFI = 0.96; RMSEA = 0.051; SRMR = 0.037). In addition, the reliability and temporal stability analyses showed acceptable indices. Based on these results, evidence of reliability and validity of the Physical Education Grit Scale in Span of Physical Education is provided.

## 1. Introduction

Physical education (PE) classes are a context where students are, at some point, confronted with a range of adverse and potentially stressful circumstances [1,2,3]. Some of the adverse circumstances present during PE lessons could be mastering new motor skills, facing fears or embarrassing situations, mastering one or more sport disciplines or facing an academic test. This allows young people to be better prepared for the demands and challenges they may face in everyday life [4,5]. In the face of these adverse circumstances present during Physical Education classes, students can develop a series of internal mechanisms that allow them to overcome the adversities they face and obtain a series of benefits, not only an improvement in relation to their mental, cognitive and social health [6,7,8], but also in achieving academic goals focused on motor skills and abilities, as well as behaviours focused on regular physical activity and long-term healthy eating [7,9,10]. Therefore, in recent years several studies have emerged (e.g., Duckworth et al. [11] and Duckworth and Quinn [12]) trying to analyse the effect of Grit, as an indicator and essential component of success and achievement. However, there is currently no evidence of scales that analyse Grit in the context of Physical Education classes in Spain. Therefore, the aim is to examine of reliability and validity of the Physical Education Grit Scale (PE-Grit) Adaptation and testing of the factorial structure of the Physical Education Grit Scale for use in secondary education in Spain.

Early studies on Grit perceived it as a construct consisting of two factors, persistence of effort and constancy of interest [13]. Persistence of effort refers to the efforts that the individual maintains over time to achieve previously set goals, regardless of the challenges and failures they encounter [14]. On the other hand, the constancy of interest refers to the concern that individuals have for achieving long-term goals [15]. Thus, Grit among PE students is necessary for them to focus primarily on the content of their training [16]. In this sense, Physical Education differs from other educational fields, mainly because of the physical component in the educational process [17]. If we measure the persistence and interest of these students, these components should not be overlooked [18]. Different studies, in a similar academic context showed that students’ motivation, passion and interests have been associated with the learning content delivered [19,20]. Thus, successful performance based on effort and determination in physical activities and sports is fundamental to student success in school in achieving goals [21]. In this sense, it is the passion with which activities are carried out in class to achieve the objectives in PE that enables students to persevere and not give up. Therefore, if they find the lessons interesting, motivating and exciting, they are more likely to be engaged and to work hard in the long run, developing stronger Grit. However, Grit must be distinguished from other similar concepts such as resilience. In this sense, resilience is the ability of a human being to overcome an adverse situation. However, being resilient does not mean the absence of pain; on the contrary, it is often a feeling that brings stress and discomfort. However, we then go through a process of reflection that drives us to move forward. Thus, resilience is a construct that we can develop as we face various challenges [22]. In contrast, grit is a cognitive response or a trait of the individual’s own personality, which tends to be stable and involves maintaining a strong commitment to the activity, despite difficulties [12].

In order to analyse Grit, two scales were initially created in a non-academic context, the original Grit (Grit-O; [11]) with 12 items and the short scale version of Grit (Grit-S; [12]) with 8 items. Subsequently, different scales were developed in the context of education, such as the Academic Grit Scale [23] and the Physical Education Grit Scale (PE-Grit) developed by Guelmami, et al., [18]. This version was developed in Arabic and tested with students enrolled in universities of sports and physical education. This scale is made up of two factors and four sub-factors: Physical Grit (physical interest and physical effort) and Academic Grit (academic interest and academic effort). In order to show evidence of the reliability and factorial validity of the PE-Grit, Guelmami, et al., [18] conducted an exploratory factor analysis and a confirmatory factor analysis; the fit indices of the exploratory factor analysis reflected four factors with eigenvalues greater than 1, a sample adequacy index of the Kaiser-Meyer-Olkin measure, KMO of 0.88. As for the confirmatory factor analysis, the authors analysed three different models, with the best fit index reflecting the model made up of two factors and their respective subfactors, i.e., Physical Grit (physical interest and physical effort) and Academic Grit; (academic interest and academic effort). For more information see Guelmami, et al. [18].

Therefore, the aim of the present study is to adapt the PE-Grit scale from the Arabic context to the Spanish context and then to the academic context of secondary education and, finally, to test the factor structure of the scale. In this way, an exploratory factor analysis, a confirmatory factor analysis, reliability analysis and a t-retest will be carried out. Finally, the predictive validity of the scale will be analysed through a linear regression analysis in order to analyse the influence of the Grit on academic performance in PE. With this tool, it is expected that PE teachers will have an effective tool to assess the Grit of students and to know about the ability, perseverance and tenacity of their students when facing the different challenges they face during PE classes.

## 2. Materials and Methods

### 2.1. Participants

There were 857 secondary school students participating in the study (411 boys and 446 girls). The students studied in various schools in the south of Spain. The mean age was 15.66 (SD = 1.64).

For the analysis of temporal stability (t-retest), 147 students participated, forming a second independent sample. The mean age was 15.31; (SD = 0.95). The students completed the instrument twice with a time span of two weeks.

The sampling method was non-probabilistic inferential based on those schools to which we had access.

### 2.2. Procedure

In order to validate the factor structure of the PE-Grit Scale for use in secondary education in Spain, it was necessary to translate the items into Spanish. The strategy followed was that developed by Hambelton [24]. This method consists of the direct and inverse translation of the items by translators specialised in the field of psychology, i.e., from English to Spanish and from Spanish to English, judging their equivalence with the original version. Once the final Spanish scale was obtained, it was necessary to adapt the items to the context of secondary school physical education. In order to do so, we had the collaboration of a group of educational psychologists with extensive experience in the field of research.

Once the items of the scale had been adapted for use in secondary education in Spain, physical education teachers from several schools were contacted to ask for their collaboration. The aim of the study was explained to the teachers and their students. Those students who decided to participate voluntarily in the study were asked to provide informed consent signed by their parents. Once informed consent was obtained from the pupils, they filled in the questionnaires at the beginning of the physical education classes with pencil and paper.

In order to carry out the present study, the approval of the Bioethics Committee (Ref. 03/2021 UALBIO) of the University of Almeria was requested. In addition, the Helsinki Declaration and the protocol of the American Psychology Associations were respected.

### 2.3. Measurements

Grit in Physical Education. In order to analyse the Grit of the students during Physical Education classes, the factor structure of the PE-GRIT was developed by Guelmami et al. [18]. The PE-GRIT is made up of 16 items that are divided into two factors: Physical Grit (physical interest and physical effort) and Academic Grit (academic interest and academic effort). Responses are rated on a Likert scale ranging from 1 to 7, where 1 is strongly disagree and 7 is strongly agree.

Academic performance in PE. To measure this variable, the students’ grades at the end of the course were used. This data was provided by the teacher. The value of the grades was based on a Likert scale ranging from 1 to 5, with 1 being fail and 5 being outstanding.

### 2.4. Data Analysis

In order to test the factor structure and determine the reliability of the PE-GRIT, the statistical programmes SPSS 25 (IBM, Armonk, NY, USA) and AMOS 21 (IBM, Armonk, NY, USA) were used. Thus, a confirmatory factor analysis (CFA) and an exploratory factor analysis (EFA) were carried out to test both the factor structure of the questionnaire, the reliability of the questionnaire was analysed through the omega coefficient and Cronbach’s alpha (internal consistency), and the temporal stability (intraclass correlation index [CCI]) was analysed, which assesses the concordance of continuous data [25]. Finally, a linear regression analysis was carried out in order to test the predictive validity of the scale.

**Figure 1:**
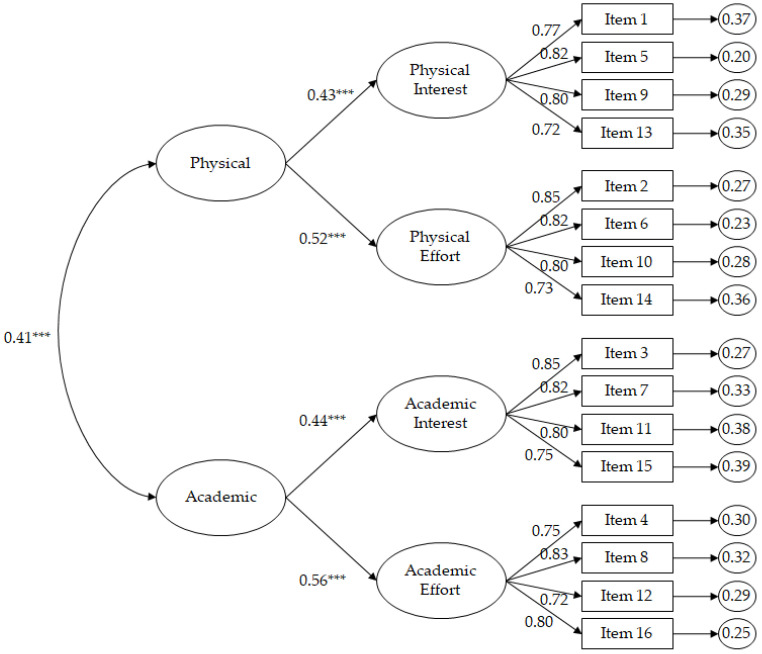
CFA of the PE-Grit focusing on the PE context. The ellipses represent the factors and the rectangles represent the different items. The residual variances are shown in the small circles. *** *p* < 0.001.

As the present study employed Likert-type scales, the maximum likelihood estimation method was used in the CFA together with a bootstrapping of 5000 interactions. The estimators were found to be robust [26]. The parameters used to reject or accept [27] the model depicted in Figure 1 were as follows: a score equal to or less than 0.06 for the RMSEA and SRMR; a score between 2 to 3 for the χ^2^/df; a score greater than 0.95 for the incremental indices CFI, IFI and TLI.

## 3. Results

### 3.1. Exploratory Factor Analysis

The results obtained in the Kaiser-Meyer-Olkin test (KMO = 0.94) and Bartlett’s statistics (χ^2^ (120) = 1167, *p* < 0.001) show acceptable fit indices. On the other hand, Table 1 shows the results obtained in the exploratory factor analysis.

### 3.2. Confirmatory Factor Analysis

The factor structure of PE-Grit revealed the following satisfactory fit indices: χ^2^ (99, N = 857) = 214.92, *p* = 0.001; χ^2^/df = 2.17; CFI = 0.96; TLI = 0.96; IFI = 0.96; RMSEA = 0.051 (90% CI = 0.042–0.061); SRMR = 0.037. Standardised regression weights were statistically significant (*p* < 0.001), ranging from 0.72 to 0.85.

On the other hand, different alternative models have been proposed (see Supplementary Material) in order to test the best fit for the scale compared to the model expressed in Figure 1. The results have shown (Table 2) that the best fit indices are those shown by the model in Figure 1 (see, Hair, et al., [27]).

### 3.3. Descriptive Statistics and Reliability Analysis

The mean, standard deviation, bivariate correlations (descriptive statistics), omega and alpha of Cronbach (the reliability analyses) and the temporal stability analysis are shown in Table 3. The results showed that each of the factors showed a good fit in terms of reliability analyses. In addition, the temporal stability analyses were adequate.

### 3.4. Linear Regression Analysis

Table 4 shows the linear regression analysis in which each of the sub-factors belonging to the Grit has been related to academic performance. This analysis tries to reflect the predictive validity of the scale, where the results have shown a positive relationship with academic performance.

## 4. Discussion

The aim of the present study is to adapt and test the factor structure of the Guelmami, et al. [18] PE-Grit to the context of secondary school PE classes in order to provide an effective tool with which to assess students’ Grit in relation to PE classes. In order to meet this objective, it was necessary to carry out several analyses, such as an EFA, a CFA, reliability and temporal stability analyses, and an analysis of criterion validity. The results of each of these analyses have shown that the PE-Grit is a valid and reliable instrument for measuring students’ perceived Grit during PE lessons.

The factor structure according to the EFA showed that the scale was composed of two factors. This result was later contrasted by the CFA. In both factor analyses the fit indices were acceptable and significant. In addition, the reliability analyses were within acceptable parameters [28]. These results were similar to those of the original scale [18]. However, the results of the temporal stability analysis, which were satisfactory [25], cannot be compared with the original scale study by Guelmami, et al., [18] as they did not perform this analysis. In this sense, the test–retest of the stability analysis showed that the students completed the scale in a similar way after two weeks. This two-week period is long enough for students not to remember the items and for there to be no change in students’ perceptions of Physical Education classes [29].

Finally, the results of the present study have shown a positive relationship of each of the subscales belonging to the Grit (physical interest, physical effort, academic interest and academic effort), with respect to academic performance. These results cannot be compared with the study of the original scale. However, the results in the study by Guelmami, et al. [18] showed that each of the scales predicted deep learning strategies, which several studies have found to be related to high academic performance [30,31]. Therefore, it can be suggested that the results of the present study are in line with the results of the original scale. Furthermore, similar studies have shown that resilience in the PE classroom has a positive influence on academic performance [7,8], while stress and anxiety have a negative influence [32]. This is because the experiences students have during PE lessons are key to students’ adherence and engagement in order to overcome adversities that may arise. Thus, if lessons are enjoyable and challenging for students and the teacher devotes time to each student, this will lead to greater student engagement, which will lead to an increase in coping strategies and thus an increase in academic achievement [33].

Despite the results achieved in the present study, there are a number of limitations that must be taken into account. The selection of participants in the study was based on non-probability incidental sampling, which hinders the replicability of the study. On the other hand, the validation of the factor structure of the PE-Grit is an ongoing process and future studies will have to endorse the results achieved in the present study. Future studies should analyse the predictive validity of the scale with respect to other variables such as motivation or engagement, which are fundamental elements for understanding the reasons for students’ perseverance in the educational system [34].

## 5. Conclusions

The results of the present study show that the PE-Grit Scale is a valid and reliable instrument to measure Grit in PE classes in a multidimensional way. This variable is essential in order to know the students’ ability to adapt to the vicissitudes they face during PE classes. In this way, it will be possible to design educational programmes that encourage adaptation and learning of educational resources that increase the number of resources and strategies for the student to better adapt to the classroom context.

## Figures and Tables

**Table 1:** Exploratory Factor Analysis.

	Physical Grit		Academic Grit	
Items	Physical Interest	Physical Effort	Academic Interest	Academic Effort
1	0.70			
5	0.77			
9	0.74			
13	0.76			
2		0.71		
6		0.75		
10		0.72		
14		0.74		
3			0.72	
7			0.70	
11			0.74	
15			0.77	
4				0.73
8				0.71
12				0.77
16				0.79

Note: Factor loadings less than 0.4 are not shown in the table. The sentence corresponding to the item can be found in Appendix A.

**Table 2:** Comparison between the different models.

Models	χ^2^/df	CFI	TLI	IFI	RMSEA	SRMR
1. Supplementary Model S1	4.18	0.92	0.92	0.92	0.078	0.065
2. Supplementary Model S2	5.88	0.88	0.87	0.88	0.10	0.066
3. Model Figure 1	2.17	0.96	0.96	0.96	0.051	0.037

**Table 3:** Descriptive statistics, reliability analysis, bivariate correlations and temporal stability analysis.

Factors	M	SD	α	ω	CCI	1	2	3	4	5
1. Physical Interest	4.15	1.31	0.81	0.81	0.90	-	0.43 **	0.42 ***	0.52 **	0.23 **
2. Physical Effort	4.79	1.23	0.83	0.84	0.89		-	0.37 **	0.39 ***	0.37 **
3. Academic Interest	3.63	1.08	0.78	0.79	0.92			-	0.42 ***	0.33 **
4. Academic Effort	3.43	1.42	0.82	0.83	0.89				-	0.46 ***
5. Academic Performance	2.67	0.37	-	-	-					-

Note: *** *p* < 0.001; ** *p* < 0.01; CCI = Intraclass Correlation Index; SD = Standard Deviation; α = Cronbach’s Alpha; M = Mean.

**Table 4:** Linear Regression Analysis.

	*F*	*R^2^*	*β*	*t*
	48.56	0.57 ***		
1. Physical Interest			0.31	1.18 **
2. Physical Effort			0.33	2.40 ***
3. Academic Interest			0.51	2.12 **
4. Academic Effort			0.46	1.56 **

*** *p* < 0.001; ** *p* < 0.01.

## Data Availability

Not applicable.

## Supplementary Materials

The following supporting information can be downloaded at: https://www.mdpi.com/article/10.3390/ijerph191610008/s1.

## Appendix A

**Table A1:** The instrument was validated in Spanish.

Physical interest	- Aunque encuentre dificultades físicas durante la sesión de entrenamiento, las considero muy importantes. - Aunque pueda hacer cosas más divertidas, no me pierdo el entrenamiento físico. - * No le doy mucha importancia a las sesiones de entrenamiento físico. - Siempre me interesan los nuevos ejercicios físicos en mis sesiones de entrenamiento
Physical Effort	- El ejercicio físico intenso nunca me desanima. - Puedo mantener un esfuerzo físico adecuado durante todo el año. - * No escatimo esfuerzos para completar el ejercicio. - Durante la práctica física, hago lo que sea necesario.
Academic Interest	- Uno de mis intereses es profundizar en los conocimientos de la asignatura, sin importar el tiempo que me lleve. - Siempre me interesa adquirir nuevos conocimientos. - * No todos los ejercicios son importantes. - Los deberes de Educacion Fisica son muy importantes para mí.
Academic Effort	- Practico fuera de clase para continuar aprendiendo. - Estoy concentrado en clase para aprender nuevas habilidades y destrezas. - *No siempre repaso todas los ejercicios. - Soy diligente en todas las actividades y ejercicios.

* The item score must be reversed.
