# Supplementary figures and images for: Adaptation and Testing of the Factorial Structure of the Physical Education Grit Scale for Use in Secondary Education in Spain
Source: Int J Environ Res Public Health. 2022 Aug 13;19(16):10008. doi: 10.3390/ijerph191610008 (PMC9408085; doi:10.3390/ijerph191610008)

## Supplementary Materials

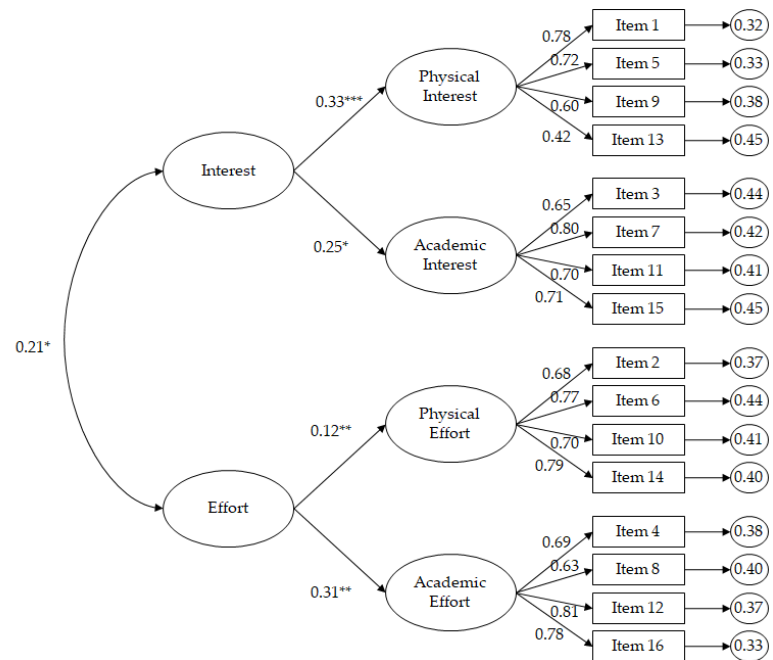

## Supplementary Model S1

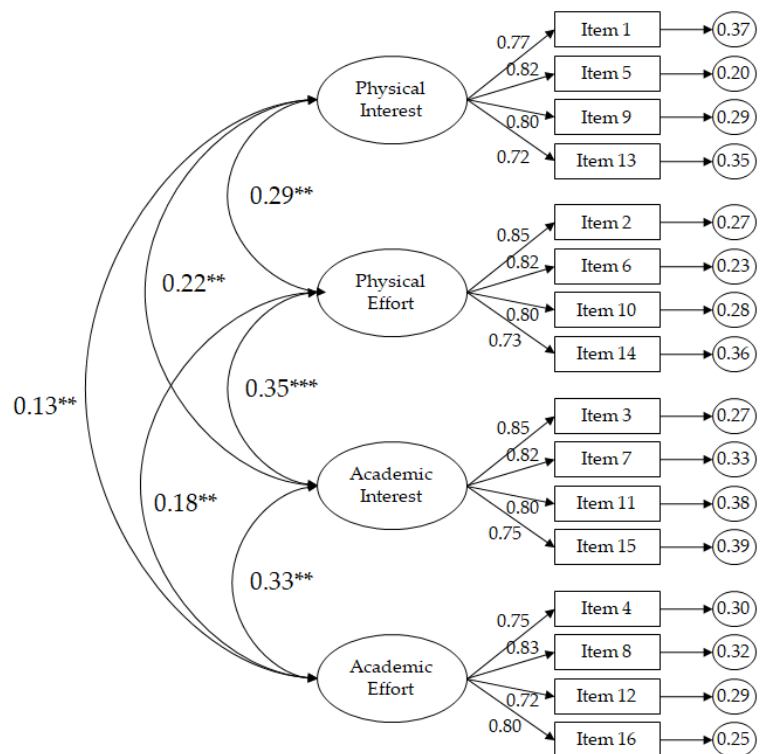

## Supplementary Model S2

Supplement: Supplementary file 1 [file ijerph-19-10008-s001.zip › ijerph-1822958-supplementary.pdf]
